# Diagnostic and Therapeutic Potential of Photo-Responsive Nanomaterials in Osteoarthritis

**DOI:** 10.14336/AD.2025.0166

**Published:** 2025-03-06

**Authors:** Yanlei Zhang, Jiachun Song, Yuxuan Li, Quanbo Ji

**Affiliations:** ^1^Medical School of Chinese PLA, Beijing, China.; ^2^School of Medicine, Nankai University, Tianjin, China.; ^3^Department of General Surgery, First Medical Center of PLA General Hospital, Beijing, China.; ^4^Department of Orthopedics, Forth Medical Center of PLA General Hospital, Beijing, China.

**Keywords:** photo-responsive nanomaterial, osteoarthritis, cartilage, photoacoustic imaging, photothermal therapy

## Abstract

Osteoarthritis (OA) is the most common musculoskeletal disease globally and is the main reason for the chronic pain and disability in people over sixty-five worldwide. Degradation of the articular cartilage, synovial inflammation and osteophyte formation are widely acknowledged as the primary pathological manifestations of OA. OA affects more than 300 million people all over the world, bringing extremely large socioeconomic burden. Unfortunately, there’s so far, no disease-modified drugs to treat it, and techniques for early detection are absent. Photoacoustic imaging is a promising imaging method based on photothermal effects, which shows enormous potential in precisely monitoring the development of OA and tracking the drug treatment progress. Photothermal therapy is a non-invasive treatment curing diseases by converting the energy from light to heat through tissue absorption. Ample research evidence verifies the efficacy of photothermal therapy in treating OA. This narrative review covered recent advances of photosensitive nanomaterials applied in OA and illustrated the potential of them in diagnosing and treating OA, hoping it could pave the way for the following theranostics and clinical transition of OA.

## 1. Introduction

Osteoarthritis (OA), the main pathological manifestation of which is the deterioration of articular cartilage, is a common and prevalent musculoskeletal disease all around the world and is the main reason for chronic pain and disability in people over sixty-five [1, 2]. Because of the irreversibility of cartilage degeneration, clinical treatments for OA mainly aim at alleviating symptoms and improving the quality of life of patients [3]. So far, the major obstacles in OA theranostics are the lack of disease-modified medicine and accurate imaging modalities that can precisely monitor the cartilage degeneration especially in the initial stage of disease. Photoacoustic imaging (PAI) is a hybrid imaging method based on photothermal effects [4], which has drawn lots of global attention during the past decade because it simultaneously combines the strengths of both ultrasonic and optical imaging modalities [5]. Given its advantages of high spatial resolution and excellent imaging contrast, PAI is demonstrated to be able to image from macro to micro fields and is widely applied in the early diagnosis and detection of multiple diseases including cancer [6, 7]. Compared to the traditional imaging modalities for OA diagnosis, for example X-ray, computerized tomography (CT), and magnetic resonance imaging (MRI), PAI is capable of providing real-time structural information and elucidating physical deficit and pain severity of the osteoarthritic joints [4, 5, 8]. Therefore, PAI shows great potential in eliminating the discrepancy of structural damage and symptomatic severity in OA patients. In addition, it has been demonstrated that PAI can not only precisely monitor the development of OA, but also track the drug treatment progress [9, 10], which exerts significant promise for its clinical implementation.

As to the treatment methods for OA, currently, pharmacological interventions, non-steroid anti-inflammatory drugs (NSAIDs) in particular, aim to alleviate the principal symptoms related to pain and inflammation [11]. No disease-modified drugs are currently available to delay the development and progression of OA [12]. Current strategies are limited to lubricate joints, alleviate inflammation, eliminate free radicals and promote cartilage regeneration [11, 13, 14]. Intra-articular injection shows remarkable strengths in treating OA; however, because of the synovial fluid and avascular cartilage structure, the fast clearance of the injected agents in joint cavity is still a vexed problem [15]. Besides, it is an invasive intervention method, which may lead to inevitable joint damage. Combining therapeutic agents with targeted and functionalized nanomaterials may offer a hopeful solution to the forementioned challenges. In Deng’s study, by disguising new poly(lactide-co-glycolic acid) nanoparticles coated in chondrocyte membranes (CM-NPs), they demonstrated the efficient adhesion and deeper penetration of the particles to cartilage and in this way realized the longer in vivo retention (at least thirty-four days) of nanoparticles in the cartilage tissue [15]. Meanwhile, photothermal therapy (PTT) is a non-invasive therapeutic method curing diseases by converting light energy to heat through tissue absorption of the light [16] and has been extensively investigated for efficacy and safety and ample research evidence has verified the therapeutic effects of nanocarriers-enhanced PTT in treating OA [17, 18].

In the review, we discussed the recent advances of the photo-responsive nanomaterials used in PAI for OA and briefly summarized the utilization of photo-responsive nanomaterials in treating OA, particularly in the application of PTT.

## 2. Utilization of nanomaterials in PAI of OA

## 2.1 Cartilage-targeted nanoparticles

**Figure 1.**
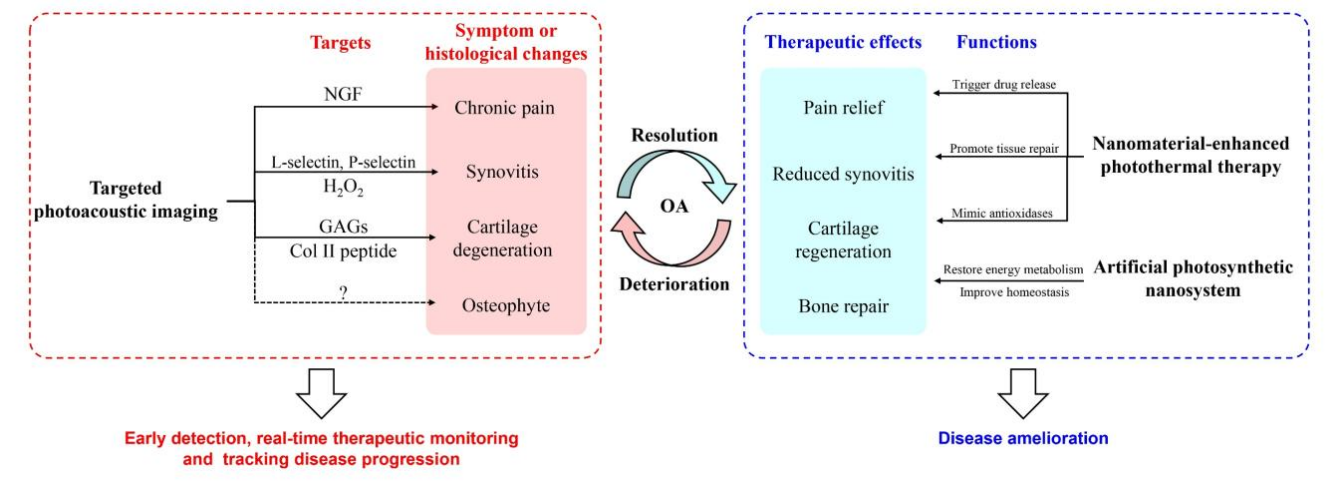
**Systematic summary of the roles of photo-responsive nanomaterials-enhanced diagnosis and therapies in osteoarthritis implementation**. Dotted line means there’s so far, no research evidence for this aspect. Abbreviations: NGF: nerve growth factor; GAG: glycosaminoglycan; Col II: type II collagen.

Cartilage degeneration is one of the hallmarks of OA (Figure 1) [19]. Early diagnosis and dynamic visualization of articular cartilage loss are necessary for timely and effective cartilage repair. However, the existing imaging modalities, for example X-ray, CT, and MRI, can just detect cartilage degeneration indirectly or in the late-stage of OA progression [20]. Molecular imaging, for instance targeting biochemical cartilage composition, has become an attractive alternative. In particular, the loss of glycosaminoglycans (GAGs), the components of articular cartilage extracellular matrix (ECM), are supposed to reflect OA development [21]. Chen et al. [9] introduced cartilage-targeted endogenous melanin nanoparticles (MNPs) encapsulated by poly-l-Lysine (PLL) nanoparticles for the PAI of cartilage deterioration in OA through the powerful electrostatic interactions between nanoparticles and the GAGs. The PLL-MNPs were found to present outstanding photoacoustic imaging (PA) intensity, low toxicity, good photostability and biocompatibility. By using PAI, researchers could clearly detect and analyze the changes of the GAG content in cartilage so that to tell apart from the early OA from the late and monitor the efficacy of drug administration in OA patients. In their following study [10], PLL-MNPs were used to track OA progression. PA signal intensity showed sequential reduction along the progression of OA, while results of X-ray showed visible joint damage signs until six weeks. Confirmed by histologic examinations, the content of GAGs in the cartilage decreased steadily along with the development of OA from the initial-point to the end-stage and the histologic findings were positively related to the PAI results [10]. That is to say, detecting GAG contents changes using PLL-MNPs-enhanced PAI provided sensitive and consistent visualization of OA development, which is of high significance for the future clinical monitoring of disease development and the therapeutic efficacy tracking.

Collagen II is the structural backbone of the ECM of articular cartilage [22], which might also be another promising target for the nanomaterial-enhanced PAI (Figure 1). Shen et al. [23] successfully fabricated a novel collagen II peptide-targeted nanoprobe for monitoring the early loss of cartilage by coating Au nanoparticles (Au@PDA NPs) with polydopamine (PDA) and immobilizing WYRGRL peptide on the surface. The enrichment of Au@PDA NPs in cartilage later led to the localized plasmon resonance coupling effect, resulting in an amplification of photothermal conversion capacity after aggregates formation [23]. Furthermore, the catechol structure in the PDA shell was shown to be able to eliminate the reactive oxygen species (ROS) and effectively delay the disease progression of OA [23].

## 2.2 Inflammation-targeted nanoparticles

Inflammation, synovitis in particular, is one of the main histopathological manifestations of OA [19, 24, 25]. In MRI-based clinical research, synovial thickening was found even in 73% of the patients at the mild end of the osteoarthritic spectrum [26], suggesting synovitis might be an indicator for early detection and evaluation of OA. Angiogenesis, hypoxia, hyperemia are vital biomarkers of synovitis [27, 28]. PAI has been shown to capably assess synovitis non-invasively by detecting increased angiogenesis and hypoxia in the synovium of mice with OA induced by destabilization of the medial meniscus (DMM) [29]. PAI is also promising in measuring the synovial angiogenesis in rheumatoid arthritis (RA), which is suggested to be more severe in synovitis [30]. In a well-established RA rat model, PAI images can not only distinguish the OA joints from the normal ones but also assess the functional changes after drug administration [31]. Combining with nano contrast agents, PAI is shown to be more targeted. Li et al. [32] reported a core-satellite gold nanoparticles (AuNPs) for surface-enhanced Raman scattering (SERS) and PAI to enable the accurate quantitative monitoring of H_2_O_2_, which was an important representative of ROS molecule, in OA rabbits. The results suggested that the combination of H_2_O_2_-activated second near-infrared (NIR-II) PAI and SERS imaging promotes the accurate differentiation between the inflamed tissue and normal area. In addition, the mesoporous silica shell of the AuNPs helped to deliver drugs to the target areas to realize the targeted intervention. Therefore, these core-satellite AuNPs can dynamically and quantitatively monitor the production of H_2_O_2_ in OA rabbits, but also track the efficacy of the anti-inflammatory treatment simultaneously [32]. Vonnemann et al. [33] synthesized a targeted imaging agent for the multispectral photoacoustic tomography of RA based on functionalized polyanionic polysulfated Au nanorods (AuNRs). The artificial AuNRs were expected to specifically bind to L-selectin of invading leukocytes and P-selectin on vascular epithelium, so that to address the inflammatory synovium. The following experiments suggested nanorods significantly reduced cytotoxicity and permitted the visualization of inflammation in RA model mice with high contrast [33]. These forementioned results pave the way for future exploration in other inflammation-associated pathologies like OA (Fig. 1).

Pain is the predominant symptom and the primary reason for OA patients to seek medical help [34]. Yet current diagnostic methods for OA pain are subjective and nonspecific. Pain is closely associated with synovitis in OA [35]. Since never growth factor (NGF) is a dominant player in pain sensation of OA, Au et al. [8] reported an NGF-targeted theranostic nanoprobe based on molybdenum disulfide (MoS_2_) nanosheet-coated AuNRs (MoS_2_-AuNRs) for photoacoustic pain imaging. The photoacoustic and photothermal performance of AuNRs were shown to be significantly improved by coating with MoS_2_. And by conjugating with NGF antibody, MoS_2_-AuNRs were functionalized and targeted the painful OA knees actively [8]. Hence, the NGF-targeted theranostic MoS_2_-AuNRs were effective to localize the source of joint pain under PAI. This research implicates the potential of the targeted nanomaterials for both molecular and functional imaging in OA (Fig. 1).

## 3. Therapeutic potential of photo-responsive nanomaterials in OA

## 3.1 Photothermal therapy (PTT)

NIR light is a promising agent in the application of disease treatment because of its properties of efficient tissue penetration depth, lower scattering and absorption [4, 36]. In addition to the conventional pharmacy and surgery applied for end-stage OA patients, applying NIR-sensitive nanomaterials in PTT for treating OA through mitochondrial repair [37, 38], cartilage protection [37, 39], inflammation suppression [18] and pain relief [8, 40] is attractive and promising (Table 1).

## 3.1.1 Photothermal-triggered drug release

Photothermal nanomaterials control drug release through their photothermal properties, and the synergy of heat and light often results in multiplying therapeutic effects [36]. NIR usually acts as a smart switch to trigger the subsequential release of drug in treating OA in order to solve the problem of unsatisfying drug clearance in OA joints. Zhao et al. synthesized a PEGylated, phenylboronic acid modified L-DOPA pro-antioxidant (pPAD) that can self-assemble into nanoparticles (pPADN) for the loading of dexamethasone (Dex) [41]. After activated by ROS, pPADN were transformed into the active form of L-DOPA, and were transformed into antioxidative melanin-like materials following a serial of oxidative reactions. The in situ structural transformation made pPADN fit for tracking the efficacy as an excellent PAI contrast agent and the structural transformation of pPADN triggered the release of Dex, which further led to mitigated synovitis, ameliorated joint destruction and remarkably reduced the degradation of cartilage matrix with almost no in vivo toxicity [41]. Another intra-articular drug delivery nanosystem, MoS_2_@CS@Dex (MCD), was proposed using the chitosan (CS)-modified MoS_2_ nanosheets [42]. In this research, the release of Dex was triggered by photothermal conversion in MCD, meanwhile the long range-controlled release of Dex into the joint was realized by modulating the NIR light irradiation. More importantly, MCD was later found to prolong the residential time of Dex in the joint cavity. Therefore, the intra-articular injection of MCD combining with NIR radiation provided a significant enhancement of Dex at low systemic doses and attenuated the cartilage erosion caused by inflammatory mediators such as TNF-α and IL-1β [42]. In addition, the PAI capability of MoS_2_ nanosheets realized the detection of MCD metabolism in joint cavity [42], which enabled the real-time *in vivo* monitoring of therapeutic efficacy. Li et al [17]. prepared a novel NIR light-sensitive drug delivery system (DS-TD/MPDA) using mesoporous polydopamine nanospheres (MPDA) as NIR responders and drug carriers, diclofenac sodium (DS) as the anti-inflammatory agent, and 1-tetradecanol (TD) as the drug administrator. Once exposed to 808 nm NIR light (power density 1.5 W/cm^2^, eight minutes per day, twice a week for two weeks), the release of DS was effectively controlled. Besides, the following histological analysis suggested that DS-TD/MPDA significantly alleviated the cartilage degeneration of temporomandibular joint (TMJ), thus ameliorating OA progression.

**Table 1:** Summary of the studies applying photo-responsive nanomaterials in photothermal therapy for treating osteoarthritis.

Study	Light parameters used in photothermal therapy				Types of nanomaterials	Main results	Involved pathways or mechanisms	Adverse effects
	Wavelength (nm)	Power density (W/cm^2^)	Irradiation time (min)	Sessions and duration				
**Chen et al, 2019^[18]^**	650	0.5	10	/	Notch1-siRNA-loaded NO-hemoglobin@siRNA@PLGA-PEG	Strongly decrease pro-inflammatory cytokine expressions and macrophage response, ameliorate inflammation and cartilage erosion.	/	No liver toxicity.
**Zhao et al, 2019^[42]^**	808	0.4	9	Four times in 8 hours	Dexamethasone-loaded chitosan-modified MoS_2_ nanosheets	Prolong the residential time of drug in the joint cavity.	Downregulating the secretion of TNF-α and IL-1β	No toxicity or side effects in the metabolism.
**Au et al, 2021^[8]^**	808	0.2	10	/	NGF-targeted MoS_2_ gold nanorods	Mitigate mechanical pain and gait. Efficiently block the peripheral pain transmission.	Stopping the binding of NGF to TrkA receptors	/
**Zhao et al, 2021^[41]^**	/	/	/	/	Dexamethasone-loaded pPADN	Mitigate synovitis, ameliorate joint destruction and remarkably reduce cartilage matrix degradation.	/	Negligible in vivo toxicity.
**Wu et al, 2022^[49]^**	/	/	/	/	gelatin-methacryloyl/poly (methyl methacrylate)/polydopamine hydrogel	The hybrid hydrogel has favorable photothermal ability, biocompatibility, and osteogenic effects.	/	/
**Xu et al, 2022^[37]^**	808	/	10	/	Epigallocatechin gallate decorated Au-Ag nano-jars	Reduce the apoptosis of chondrocytes, facilitate cartilage migration and regeneration repair.	/	/
**Li et al, 2023^[17]^**	808	1.5	8	Twice a week for two weeks	Diclofenac sodium-loaded mesoporous polydopamine nanospheres	Significantly alleviate cartilage deterioration and delay disease progression.	/	Few toxic side effects during metabolism.
**Shi et al, 2023^[12]^**	808	0.3	10	/	Molybdenum-based polyoxometalate nanoclusters	Effectively upregulate antioxidant enzymes, alleviate pain, diminish inflammatory responses, reduce catabolic proteases, and mitigate disease progression.	/	/
**Yu et al, 2023[14]**	/	/	/	/	MPMP	Eliminate ROS and RNS and supply oxygen, promotes the chondrogenesis in inflammatory conditions.	Deactivating NF-κB/IL-17 signaling pathways and enhancing MAPK signaling pathway	/
**Li et al, 2024^[40]^**	880	0.75	20 s interval with 15 s irradiation as one cycle and three consecutive cycles each time	Once a week for twelve weeks	Citrate-stabilized gold nanorods	Significantly activate TRPV1, remarkably attenuate cartilage degradation, osteophyte formation and subchondral bone sclerosis. Physical activities and pain are improved.	Hampering ferroptosis in chondrocytes.	No visible toxicity.
**Yang et al, 2024^[38]^**	808	/	15	/	PtCuO_x_/CeO_2-x_ nanozyme	Effectively scavenge intracellular ROS and RNS, inhibit inflammatory factors, and thus reduce chondrocyte apoptosis.	Inhibiting the ROS/Rac-1/NF-κB signaling pathway	Good biosafety in vivo.

Nitric oxide (NO), a bioactive molecule, can be used to treat arthritis by suppressing inflammation and inhibiting pro-inflammatory factors [43, 44]. In order to accurately control the release of NO, Chen et al. [18] constructed a NO nanogenerators, NO-hemoglobin@siRNA@PLGA-PEG (NHsPP), by assembling photothermal-agents and NO molecules within nanoparticles, and loaded the new nanoparticles with Notch1-siRNA to achieve precise efficacy. In the NHsPP, the hemoglobin nanoparticles acted as the NO carrier, and converted the absorbed NIR light energy into heat to trigger the release of NO. Given the synergistic effects of PTT, the NHsPP achieved simultaneous intervention with NO, siRNA and PTT. In vitro experiment results suggested the combination therapy strongly decreased the expressions of pro-inflammatory cytokines and curbed the response of macrophages. Meanwhile, the NHsPP accumulated effectively in OA mice, and significantly ameliorated the joint inflammation and cartilage destruction without any liver toxicity [18].

## 3.1.2 Targeted photothermal-responsive nanoparticles for treating OA

Gold nanoparticles have been widely used in PTT. Using NGF-targeted MoS_2_ gold nanorods (MoS_2_-AuNRs), NIR light (wavelength 808nm, power density 0.2 W/cm^2^, irradiation duration 10 minutes)-excited PTT could mitigate mechanical pain and gait for both subacute and chronic OA [8]. This molecular theranostic method is demonstrated to be able to efficiently block the peripheral pain transmission through stopping the conjunctions of NGF to TrkA receptors [8]. Activation of transient receptor potential vanilloid 1 (TRPV1) is widely investigated to contribute to the alleviation of cartilage degeneration [45, 46]. Li et al. [40] made the Citrate-stabilized gold nanorods (Cit-AuNRs) by conjugating to the TRPV1 monoclonal antibody (Cit-AuNRs@Anti-TRPV1) as a photothermal switch for TRPV1 activation in chondrocytes upon exposure to NIR. Intra-articular injection of Cit-AuNRs@Anti-TRPV1 followed by NIR irradiation (wavelength 880 nm, power density 0.75 W/cm^2^) resulted in significant activation of TRPV1 and attenuation of cartilage degradation by hampering ferroptosis in chondrocytes. The osteophyte formation and subchondral bone sclerosis were also remarkably ameliorated. Furthermore, physical activities were improved, and pain of DMM-induced OA mice was relieved through activating TRPV1 induced by Cit-AuNRs@Anti-TRPV1 [40]. Through integrating the interference oligonucleotides with the AuNRs, Chen et al. [39] constructed the spherical nucleic acids (SNAs) aiming to regulate inflammation and cartilage degeneration related to abnormal IL-1β mRNA expression. After loading the AuNRs with SNAs, the fabricated (DNA)HA-SNAs system (HA-SNAs) exhibited a reversible NIR-triggered on-demand release of SNAs by photothermal-induced DNA dehybridization and post-NIR in situ hybridization. Both in vitro and in vivo experiments showed that the effects of this therapeutic nanosystem in down-regulating catabolic proteases and up-regulating anabolic components in cartilage over extended periods, and in this way to safeguard the chondrocytes against degeneration and to further impede the progression of OA [39]. In conclusion, AuNPs are promising targeted photothermal-responsive agents for treating OA. However, when applying AuNPs, it’s essential to seriously consider the size-dependent cytotoxicity of AuNPs in chondrocytes [47].

Hydrogels are commonly applied in treating OA and when combined with nanomaterials, for example polydopamine nanoparticles, which exhibit excellent absorptions at infrared wavelengths, these nanocomposite hydrogels are demonstrated to be used as options for the application of PTT. Injectable hybrid hydrogels have been widely explored in the local PTT of cancer, and they are found to be capable of effectively eradicating tumor with little damage to adjacent tissue [48]. In Wu’s research, they formulated a gelatin-methacryloyl/poly(methyl methacrylate)/polydopamine (GelMA/PMMA/PDA) hydrogel. This hybrid hydrogel was demonstrated to have favorable photothermal ability, biocompatibility, and osteogenic effect. When combined with mild PTT, the GelMA/PMMA/PDA hydrogel possessed better bone repair compared to the controls suggesting the beneficial osteogenic ability and providing a novel approach to effectively promote bone repair and tissue regeneration [49].

## 3.1.3 Nanoenzymes

ROS play important roles in the onset and development of OA and antioxidant enzymes are reduced in the context of OA, which will in turn exacerbate oxidative stress cascades [50]. Therefore, ROS might be a promising target for OA treatment. Molybdenum-based polyoxometalate (POM) nanoclusters exert excellent NIR-responsive properties and prove to be potential candidates for ROS-associated diseases [51]. Under NIR exposure, the ROS scavenging activity of POM nanoclusters was established to be enhanced, suggesting the NIR-responsive POM nanoclusters were novel excellent nano-antioxidants for protecting OA joint [12]. In addition, NIR-responsive POM (wavelength 808 nm, power density 0.3 W/cm^2^, ten minutes per time) effectively upregulated antioxidant enzymes, alleviated OA pain, diminished inflammatory responses, reduced catabolic proteases, and attenuated the progression of OA [12]. All these above results indicated the splendid antioxidant and anti-inflammatory effects of NIR-responsive POM. Yu et al [14] constructed a dual-bionic photothermal nanozyme (MPMP) to mimic antioxidases and hyaluronan synthase for OA therapy. The antioxidases-mimicking properties of MPMP nanozyme contributed to eliminating ROS and reactive nitrogen species (RNS) and supplying oxygen. Irradiated by NIR light, the MPMP nanozyme triggered thermogenesis and the release of Mg^2+^, which promoted chondrogenesis in inflammatory conditions by deactivating NF-κB/IL-17 signaling pathway and meanwhile enhancing MAPK signaling pathway [14]. Cerium oxide (CeO_2_) nanospheres have an "oxidation switch" to promote catalytic activity through adding oxygen vacancies. Yang et al. [38] developed PtCuO_x_/CeO_2-x_ nanozymes and combined it with NIR irradiation to regulate the microenvironment for OA therapy. The nanozymes had excellent photothermal conversion efficiency. Furthermore, the PtCuO_X_/CeO_2-X_ was of highly efficient SOD/CAT-like activity to effectively scavenge intracellular ROS and RNS, inhibit inflammatory factors, and thus reduce chondrocyte apoptosis. These anti-OA effects were later demonstrated by *in vivo* experiments to be further enhanced through NIR irradiation. Mechanistically, PtCuO_x_/CeO_2-x_ nanozymes decreased the expressions of Rac-1 and *p*-p65 as well as ROS and remodeled the inflammatory microenvironment of OA joint by curbing the ROS/Rac-1/NF-κB signaling pathway [38].

Another Epigallocatechin gallate (EGCG) decorated Au-Ag nano-jars (E@Au-Ag) were applied as NIR-sensitive nanoenzymes for treating OA by enhancing mitochondrial repair and cartilage protection [37]. In this study, E@Au-Ag possessed intrinsic properties of antioxidation and reduced the apoptosis of chondrocytes. Under NIR irradiation (808 nm for ten minutes), the release of EGCG promoted the regeneration of articular cartilage. The subsequential results suggested that E@Au-Ag ameliorated cartilage destruction which was evaluated through decreased OARSI score after eight weeks of treatment in OA animals [37]. Therefore, combining the multifunctional nanoplatform with NIR irradiation facilitates cartilage repair and provides a novel promising OA therapeutic strategy.

## 3.2 Nanomaterial-based photosynthetic system for treating OA

Recently, Chen et al. [52] developed a novel therapeutic strategy for OA. In their innovative research, a natural, nanosized plant-derived photosynthetic system based on nanothylakoid units (NTUs) was prepared, which was later demonstrated to be capable of controllably enhancing cell anabolism by independently providing key energy and metabolic carriers. To enable cross-species applications, they used the chondrocyte membrane (CM) as the camouflage encapsulation. They demonstrated that these CM-NTUs entered chondrocytes through membrane fusion, which ensured less lysosome degradation and rapid penetration. Moreover, the CM-NTUs increased the expressions of ATP and NADPH in situ after being exposed to light and improved anabolism in degenerated chondrocytes [52]. The CM-NTUs could also systemically reverse the imbalance of energy metabolism and restore cellular metabolism to maintain cartilage homeostasis and protect against disease progression of OA.

## 4. Conclusion and perspectives

Nanotechnology is an innovative force introducing creative solutions to the limitations of conventional theranostic methods in diagnosing and curing various diseases including cancer and OA. In this narrative review, we have reviewed the application of photo-responsive nanomaterials in the diagnosis and treatment of OA, especially in the field of PAI and PTT. PAI is shown to be an excellent alternative for early detection and real-time therapeutic monitoring for OA. While PTT exerts great potential in treating OA not only because it’s a non-invasive interventional method, but also because the bright perspectives induced by combining it with targeted nanomaterials. Despite the current advances in preclinical research, obstacles still persist.

While the therapeutic promise of photo-responsive nanomaterials in OA is evident from current studies, their long-term biocompatibility and systemic toxicity profiles require further rigorous investigation. For non-degradable metal-based nanomaterials (e.g., AuNPs, TiO_2_), prolonged retention in joint tissues may induce oxidative stress through inflammatory reactions or mitochondrial dysfunction [53]. Conversely, biodegradable polymers (e.g., PLGA-based systems) could lead to cumulative inflammatory responses due to acidic degradation byproducts altering synovial pH microenvironments [54, 55]. More detailed and targeted strategies should be taken into consideration while designing them *in vivo* experiments and clinical trials. Besides, medical translation from lab to clinic, large-scale randomized controlled trials in particular, is urgently needed because the examples of applying nano contrast agents for PAI and PTT in OA patients are still rare till now.

To address these concerns, we propose a dual-strategy framework for future studies: (1) Toxicity-by-design optimization: Incorporating real-time toxicity sensors (e.g., ROS-responsive fluorescence probes) or "fail-safe" mechanisms (e.g., auto-catalytic degradation upon exceeding safe nanoparticle concentrations); (2) Multi-scale biodynamic modeling: Utilizing machine learning to predict long-term biodistribution patterns based on nanoparticle hydrophobicity, surface charge, and patient-specific factors (e.g., renal clearance efficiency in elderly OA populations). Regulatory agencies like the FDA have emphasized such mechanistic toxicology data in nanotherapeutic guidelines [56], underscoring the urgency of this research direction.

In addition, although knee OA is most prevalent and receives the most exploration and investigation, it’s still not feasible to generalize the experience of diagnosis and treatment of knee OA to other kinds of OA, for example, TMJ OA and hip OA.
